# Ambivalent acceptance of active exoskeletons in long-term care: between perceived task-specific support and practical constraints

**DOI:** 10.3389/fdgth.2026.1891618

**Published:** 2026-10-07

**Authors:** Sebastian Hofstetter, Pascal Müller, Dominik Behr, Katrin Gayen, Patrick Jahn, Stefan Dowiasch

**Affiliations:** 1 Department of Internal Medicine, University Medicine Halle (Saale), Health Service-Research Working Group, Acute Care, Martin-Luther-University Halle-Wittenberg, Halle (Saale), Germany; 2 Dorothea-Erxleben-Learning-Centre, Faculty of Medicine, University Medicine Halle (Saale), Martin-Luther-University Halle-Wittenberg, Halle (Saale), Germany; 3 Department of Nursing, Midwifery and Therapeutic Sciences, Healthcare & Medical Technologies, Bochum University of Applied Sciences, Health Campus, Bochum, Germany

**Keywords:** assistive technology, caregivers, digital assistive technology, exoskeleton, long-term care, nursing, occupational health, usability

## Abstract

**Background:**

Lower back pain (LBP) is prevalent among nursing staff in long-term care facilities. This condition has been linked to repetitive resident handling, sustained non-neutral postures, and cumulative physical workloads. Despite growing interest in exoskeletons as ergonomic and digital assistive technologies (DAT), evidence regarding the implementation of active exoskeletons in real-world nursing practice remains limited.

**Objective:**

The objective of this exploratory mixed-methods feasibility study was to evaluate the implementation of the HAL® Lumbar Type active exoskeleton in long-term care settings. Specifically, this study explored feasibility, usability, nurses’ acceptance, and descriptive pain-related outcomes under real-world conditions.

**Methods:**

A non-randomized exploratory mixed-methods feasibility study was conducted in three long-term residential care facilities in Germany. The HAL® Lumbar Type was introduced for use in routine nursing practice during a 90-day intervention period. Objective utilization and adherence data were not systematically recorded. Quantitative outcomes (*n* = 15) included subjective LBP, functional impairment, and health-related quality of life measured using the visual analog scale (VAS), Oswestry Disability Index (ODI), and 8-item Short Form Health Survey (SF-8), respectively. A qualitative semi-structured interview study (*n* = 9) explored participants’ experiences of usability, workflow integration, and acceptance. Qualitative data were analyzed using systematic text condensation.

**Results:**

The findings suggested a pattern of ambivalent acceptance. Caregivers reported situational support during forward-leaning and transfer-related activities, particularly through perceived postural stabilization. The aggregated unpaired VAS data showed no descriptive group-level reduction in subjective pain, and individual pain trajectories could not be assessed. Some participants had lower ODI scores at follow-up, whereas the SF-8 trajectories were heterogeneous and partially indicated a lower health-related quality of life. Participants described spatial limitations, device weight, thermal discomfort, restricted mobility, and the time required for donning and adjustment as barriers to routine use. Participants reported using the exoskeleton selectively for specific high-load tasks rather than continuously throughout shifts.

**Conclusion:**

The active lumbar exoskeleton was perceived to provide task-specific ergonomic or postural support during selected physical care activities. However, participants’ accounts suggested that the perceived practical viability of the device and their willingness to use it may be associated with workflow compatibility, ease of use, perceived fit, and organizational conditions. Long-term adoption beyond the intervention period was not assessed. Future advancements in exoskeleton technology should prioritize lightweight designs, customized fits, and seamless integration strategies into dynamic nursing workflows. Larger longitudinal studies with appropriate comparison groups and objective biomechanical measures are needed to determine the clinical effectiveness and further examine implementation conditions in nursing practice.

## Introduction

Lower back pain (LBP) is one of the most prevalent work-related musculoskeletal disorders among nursing personnel, with a particularly high prevalence in long-term care facilities (1–3). While physically demanding activities such as patient handling and sustained postures are present across different care settings, available data indicate that certain types of physical strain occur more frequently during long-term care. In particular, work in constrained postures (e.g., bending, kneeling, or crouching) is reported more often in long-term residential care settings than in acute care settings (4, 5). Within this context, work in long-term care is not only characterized by repetitive resident handling and sustained non-neutral postures but also by extensive walking distances accumulated throughout daily care routines. As these activities are performed continuously and repeatedly for the same residents over prolonged periods, they contribute to a cumulative physical workload that differs from the more episodic and variable demands that are typically observed in acute care settings (6). This distinction is particularly relevant in long-term care environments, where physical activity is embedded in repetitive occupational tasks, constrained postures, and resident handling demands, rather than autonomous health-promoting movements (7, 8).

Despite the implementation of various conventional ergonomic approaches, including educational interventions focused on spinal health and self-management strategies (9), sustaining the impact of these measures on the daily occupational burden and long-term musculoskeletal health has often been described as challenging in complex care settings (3). While Ghadyani et al. (9) demonstrated that multidisciplinary education can effectively reduce pain intensity in the short term, the practical integration of such behavioral changes remains constrained by the persistent physical demands of the nursing environment. In this context, wearable assistive systems, more commonly referred to as exoskeletons, have garnered increasing attention as novel approaches for reducing mechanical strain (10–12). Nonetheless, extant research has primarily focused on industrial applications and biomechanical investigations. Another limitation of previous research is its predominant focus on short-term laboratory- (13) or simulation-based studies (14–16).

Passive exoskeletons store and release energy through mechanical components such as springs or elastic structures and have been investigated considerably more frequently than active systems. In different healthcare settings, passive exoskeletons have demonstrated the potential to reduce biomechanical loading during patient handling and other physically demanding tasks. However, healthcare professionals have also reported limitations related to restricted mobility, heat accumulation under protective clothing, pressure points caused by mechanical design, and reduced compatibility with dynamic nursing workflows (15–17). Field studies further showed that passive exoskeletons are typically worn selectively during specific high-load activities rather than continuously throughout the entire work shift (12).

In contrast, active exoskeletons provide adaptive assistance through motorized actuation and intention-based control mechanisms. Owing to fundamental differences in technical design, control mechanisms, and human-machine interactions, findings from studies on passive exoskeletons cannot be directly transferred to active systems. Despite their theoretical advantages, empirical evidence regarding the use of active exoskeletons in nursing remains scarce. Evidence from prolonged real-world implementations in long-term care settings is limited (13, 16, 18).

Despite the documented biomechanical benefits of passive and active exoskeletons, there is a paucity of evidence supporting their widespread integration into standard nursing practices. A challenge in implementing these technologies has been characterized as the “dilemma behind the product” (19). This term refers to the observation that many assistive systems remain underutilized, despite their demonstrated ergonomic benefits. This underutilization is attributed to two main factors: insufficient usability and integration into complex daily workflows, and a lack of social acceptance.

Against this background, the present study addresses this implementation gap by integrating and evaluating an active exoskeleton, the HAL® Lumbar Type, under real-world conditions in long-term care over a 90-day intervention period (18). The HAL® Lumbar Type utilizes bioelectrical control to regulate an active exoskeleton system, which uses actuators – typically electric motors – but also permits the integration of alternative drive systems (14, 20). This dynamic human-machine interaction enables a more customized and context-sensitive form of assistance during physically demanding caregiving activities (15, 17, 21, 22). In contrast to the controlled laboratory environments typical of most studies in this field, the present study was conducted under real-world conditions in long-term residential care facilities. The protracted intervention duration facilitated the exploration of caregivers’ experiences with the implementation of an active exoskeleton under real-world conditions over an extended period and the examination of perceived opportunities and barriers to its integration into routine care. This approach diverges from previous laboratory-based studies in that it assesses implementation under real-world conditions over an extended intervention period rather than during short-term simulated tasks.

### Objective

According to the study protocol, the objective of this publication is twofold: first, to present the results of this intervention study assessing the feasibility of implementing the HAL® Lumbar Type exoskeleton in long-term care settings; second, to explore the implementation of the device across four complementary dimensions: feasibility, usability, caregiver acceptance, and descriptive pain-related outcomes assessed using the visual analog scale (VAS). The study was not designed to test causal effects on pain, biomechanical load, ergonomic risk, or the prevention of work-related musculoskeletal disorders.

## Materials and methods

### Study design

This study was designed as a non-randomized, exploratory, and interventional study and fell within the feasibility and piloting phase according to the Medical Research Council (MRC) framework for complex interventions (23, 24). The methodological approach employed a sequential explanatory mixed methods design (25). Quantitative data were collected for two purposes: first, to characterize potential outcome trajectories, and second, to estimate design parameters for future confirmatory studies. The primary objective was not to demonstrate clinical effectiveness. This approach facilitated the evaluation of both quantifiable outcomes, including changes in self-reported LBP, and contextual factors associated with the implementation and utilization of technology in routine care practices.

### Setting

This study was conducted at three long-term residential care facilities providing long-term nursing care in the cities of Bitterfeld-Wolfen (Saxony-Anhalt), Witten, and Bochum (North Rhine-Westphalia). Care activities were performed in residents’ rooms, which were often spatially constrained and required flexible and adaptive movement patterns. These settings were selected to reflect the practical realities of long-term care and allow for the evaluation of the exoskeleton within everyday nursing workflows rather than under controlled or simulated conditions. This approach facilitated the evaluation of the feasibility, usability, and acceptance of the technology within a setting characterized by complex and physically demanding care procedures.

### Participants and recruitment

Recruitment followed a non-probabilistic sampling strategy based on snowball sampling, and collaborative networks were established (18). First, facility managers were informed of the study’s objectives and asked to gauge the general interest of their caregiving staff. Second, to ensure nurses’ voluntary participation and prevent management-driven exclusivity, a participatory shared decision-making approach was implemented. This process involved onsite visits by the research team to provide comprehensive briefings and allow caregivers to express their interests or concerns (18). To minimize potential bias from premature exposure to technology, these preliminary visits were strictly limited to information sessions and did not include exoskeleton testing. The final selection of the participating facilities was based on a consensus reached during consultations between the researchers and caregiving staff. This collaborative agreement was essential to ensure feasibility by securing broad support within the teams. Consequently, the study prioritized settings in which caregivers expressed a clear, voluntary commitment to integrating the HAL® Lumbar Type into their daily routines. The objective of this recruitment procedure was to ensure informed and voluntary participation while reflecting the heterogeneity of real-world caregiving environments.

The participants were caregivers employed in long-term care facilities who regularly engaged in physically demanding tasks, including patient transfers, repositioning, and mobility assistance. The inclusion criteria were as follows: first, an age of at least 18 years; second, self-reported LBP that had been previously diagnosed by a physician, had been perceived as problematic for a minimum period of three months, and had been aggravated by work-related physical demands (18). In addition, the ability and willingness to wear the exoskeleton during work shifts throughout the study period were prerequisites for participation.

The inclusion and exclusion criteria were based on caregivers’ standardized self-disclosure during the recruitment process. Owing to strict data protection regulations regarding sensitive health data and the pragmatic nature of this real-world study, direct access to medical records or external medical verification was not feasible. However, the reliability of the self-reported data was supported by the fact that all the participants performed their regular nursing duties at the time of inclusion. Given that participants on active sick leave were ineligible, a baseline level of work-related fitness was ensured. Regular contact with participants and the collection of pain-related data were intended to identify newly occurring exclusion criteria; these procedures should not be understood as systematic adverse-event or near-miss monitoring. However, the absence of medical record access and external clinical verification prevented the stratification of participants according to specific LBP diagnoses, comorbidities, medication use, and symptom duration beyond the self-reported eligibility criteria. This pragmatic approach was found to be feasible under routine care conditions; however, the clinical interpretation of pain-related outcomes was limited.

The sample size was determined based on pragmatic considerations and established methodological recommendations for pilot and feasibility trials. These estimations were strictly intended to inform sample size and power calculations for future definitive trials under real-world conditions, rather than to confirm causal intervention-related efficacy. In addition, Whitehead et al. (26) proposed that for feasibility studies not aimed at proving superiority, a sample size of 10–15 per arm is optimal for estimating the design parameters for subsequent definitive trials.

Despite the study’s lack of statistical power for inferential efficacy testing, statistical analysis offered substantial value through the following means: the primary objective was to establish baseline variability for future power calculations under real-world conditions. In addition, the identification of structural measurement challenges such as the floor effect in functional impairment is essential. Finally, the facilitation of mutual illumination between the data strands is crucial for providing a quantitative framework to contextualize the qualitative findings on usability and acceptance. This methodological approach facilitated a descriptive examination of outcome trajectories under real-world conditions and the estimation of parameters for future confirmatory studies.

### Intervention: HAL® Lumbar Type

The intervention entailed the implementation of the HAL® Lumbar Type exoskeleton (model HAL-BB04, Cyberdyne Inc., Tsukuba, Japan), a battery-powered wearable assistive device designed to provide support to the lower back during physically demanding activities (20, 27). The system facilitates the discernment of the user’s movement intentions, thereby enabling the provision of adaptive support that aligns with natural movement patterns. The device offers five adjustable levels of support and has a battery life of approximately 4.5 h, with a charging time of approximately 2 h. The exoskeleton is distinguished by its compact design and weight of approximately 3.1 kg, including that of the battery. These characteristics are intended to support use over extended periods. However, actual wearing time and fatigue associated with prolonged use were not objectively assessed in the present study. The product was certified according to the ISO 13482 standard and protected against dust (IP5X) and splashing water (IPX4) (14, 20).

For the present study, the HAL® Lumbar Type was available in sizes S and M. Prior to the intervention, trained facilitators selected the device size and checked the positioning and technical fit for each participant according to the manufacturer’s specifications as part of the sensitization, evaluative introduction, qualification, and implementation (SEQI) framework. These inspections were conducted to ensure proper positioning, operational functionality, and safety during device use. However, the study did not include systematic records of participant-level BMI, waist circumference, abdominal circumference, device size allocation, or specific fitting problems. Therefore, the anthropometric fit was technically verified but not analytically evaluated. The SEQI education concept, a structured and guided education model, was used to prepare participants for the intended integration of the digital assistive technology into nursing practice (28). The objective of this training was twofold: to ensure both comfort and functionality and to ensure safe use and optimized movement patterns for mechanical support. Furthermore, the participants were instructed on the management of the system’s technical features, encompassing fundamental troubleshooting methodologies during routine operations. As part of the training sessions, the participating caregivers were provided with contact information for Cyberdyne and two project staff members (SH and SD). This was done so that caregivers could reach a contact person at any time during the study period if they had questions or encountered problems. Adverse events and near misses were not prospectively and systematically monitored using a standardized assessment instrument, checklist, or dedicated event log. Consequently, information on potential adverse events and near misses was limited to events spontaneously reported by participants through these communication channels.

This study followed a structured longitudinal procedure consisting of three consecutive phases. The pre-intervention baseline phase (T1) was conducted over 14 days. During this phase, baseline LBP data were collected to establish individual reference values under routine working conditions. Subsequently, the intervention phase (T2) ensued, wherein the HAL® Lumbar Type exoskeleton was incorporated into quotidian care activities over 90 days. In accordance with the published study protocol, participants were instructed to use the device during work periods characterized by elevated physical exertion, particularly during morning shifts from 6:00 a.m. to 10:00 a.m. and evening shifts from 6:00 p.m. to 10:00 p.m. However, actual adherence to this intended implementation was not objectively assessed (18). No objective exposure or utilization data were systematically collected. Specifically, individual wearing time, the number of shifts during which the device was used, task-specific use, overall frequency of use, work-cycle structure, and the frequency of donning and doffing were not recorded. Consequently, adherence to the intended implementation and actual utilization patterns could not be objectively assessed.

Participants were instructed to use the exoskeleton during specific physically demanding tasks associated with lumbar strain, including lifting and repositioning residents, assisting with transfers, such as bed-to-chair movements, and performing activities in forward-bending postures, for example, while applying compression stockings. During this phase, data collection continued along with the use of the device, allowing for repeated measurements during the ongoing work processes. In addition to continuous LBP assessments, further measurements were performed at predefined time points during the intervention period. The post-intervention phase (T3) was conducted after the intervention. At this stage, the researchers performed the final quantitative assessments (SD and SH) and collected qualitative data (SD, KG, and SH). The purpose of the qualitative data was to capture the participants’ experiences with the exoskeleton.

### Quantitative data collection

The collection of quantitative data focused on the evaluation of subjective LBP, functional impairment, and health-related quality of life. The primary pain-related outcome was self-reported LBP, assessed using the VAS. The participants were instructed to repeatedly record their pain levels during the study period. This included a baseline phase, in which the participants continued their usual work routines without the use of an exoskeleton, and an intervention phase, in which measurements were intended to be taken before and after work shifts or selected care activities during which participants were instructed to use the exoskeleton. However, the measurements could not be systematically linked to individual device exposure. This repeated assessment was originally intended to capture variations in pain perception over time. Secondary outcome measures included functional impairment and health-related quality of life. Functional status was assessed using the Oswestry Disability Index (ODI) (29, 30), a widely used instrument for evaluating limitations associated with LBP. The measurement of health-related quality of life was conducted by means of the Short-Form Health Survey (SF-8) (31, 32). The instruments were administered longitudinally across the defined study phases (T1–T3), including at baseline, mid-study, and after the completion of the intervention phase. This approach enabled the evaluation of changes throughout the study.

### Qualitative data collection

A semi-structured interview guide was developed by a multidisciplinary research team following Helfferich’s SPSS approach (collecting, checking, sorting, and subsuming) for qualitative interview guide development (33). The generation of preliminary interview questions was initiated through a collaborative brainstorming session among the research team, informed by the study objectives, existing literature, and the researchers’ extant knowledge of exoskeleton implementation. The resulting questions were reviewed for openness, clarity, and neutrality. The questions were then organized into thematic domains and condensed into a semi-structured interview guide. The guide encompassed four overarching subjects: perceived benefits, barriers, usability, and workflow integration. It commenced with open-ended introductory inquiries followed by optional probing questions. The qualitative data collection was conducted to complement the quantitative findings and to gain in-depth insights into caregivers’ experiences with the HAL® Lumbar Type exoskeleton in daily care practice (18).

In accordance with the sequential explanatory mixed-methods design, the data were collected within two weeks after the finalization of the intervention period to capture reflections on the use of the device over time and further contextualize the observed outcomes. Data were collected through semi-structured interviews with a purposively selected subsample of participating caregivers. Participants for the qualitative interviews were selected from the study population using a criterion-based approach, focusing on caregivers who completed the intervention and were able to provide detailed reflections on their experiences with the exoskeleton. The participants were encouraged to openly describe their experiences. The domains encompassed perceived physical strain and alterations in the workload, usability, and comfort of the device. Additionally, these domains encompassed the overall user experience as well as perceived benefits, limitations, and suggestions for improvement.

The interviews were conducted by three members of the research team (SH, KG, and SD), all of whom had prior experience in qualitative health-service research and semi-structured interviews. The interviewers were not involved in the participants’ daily work environments and had no prior relationship with them. The interviews were audio-recorded using a PHILIPS Voice Tracer® and subsequently transcribed. The transcription process was executed using Whisper® (Open AI 2022), model variant “small” (∼244 million parameters), ASR mode “precise,” in conjunction with noScribe 0.6.2 (34, 35). Subsequently, the raw transcripts were independently cross-checked against the original recordings by three researchers (SH, SD, and PM) and cleaned according to the rules of Dresing and Pehl (36). The interviews were conducted in German and translated into English for reporting purposes (37). The research team performed translations to preserve the original meaning of the participants’ statements (38, 39).

### Data analysis

Quantitative data were analyzed using SPSS Statistics (version 31.0; IBM Corp., Armonk, NY, USA). Contrary to the study protocol (18) but in strict adherence to the CONSORT extension guidelines for pilot and feasibility trials (40), formal hypothesis testing and the calculation of inferential *p*-values were omitted because the study was not sufficiently powered to test for intervention efficacy. Instead, the analysis focused on parameter estimation to inform future trials. First, fine-grained, shift-by-shift reporting of the VAS for LBP by the participants did not occur with sufficient quality and frequency as originally intended. To maintain rigorous scientific standards, detailed longitudinal VAS analyses and all inferential statistics related to the VAS were excluded from result reporting. Therefore, the VAS data were analyzed strictly descriptively as aggregated, unpaired data using means and standard deviations. Given that the baseline and intervention phase measurements could not be linked at the individual level, individual pain trajectories and within-subject changes could not be assessed. Second, the outcomes for functional impairment (ODI) and health-related quality of life (SF-8) were reported descriptively using means, standard deviations, and mean differences accompanied by 95% confidence intervals. Furthermore, to quantify the magnitude of the changes while explicitly accounting for the limitations of the small sample size, standardized effect sizes were calculated using Hedges’ g. Hedges’ g applied a necessary mathematical correction factor to Cohen’s d, effectively removing the systematic upward bias commonly observed in sample sizes of *n* < 20, thereby providing a more accurate parameter estimate for future research (41). Furthermore, a visual inspection of individual longitudinal trajectories (*spaghetti plots*) was performed to explore interindividual heterogeneity, particularly for participants showing subjective functional improvements or quality-of-life changes. Missing values were designated as “999” and subsequently excluded from the descriptive interpretation.

Qualitative data were analyzed using the systematic text condensation (STC) method (42) with MAXQDA Analytics Pro (including AI Assist). The MAXQDA AI Assist function was used exclusively as a supportive tool to facilitate data organization and generate preliminary coding suggestions. A critical review, discussion, and subsequent acceptance, modification, or rejection of all AI-generated suggestions were conducted by the researchers (SD, SH, PM, and KG). The identification of codes, category development, thematic synthesis, and final interpretation of the data were performed exclusively by the research team following Malterud’s Systematic Text Condensation (STC). Following multiple readings, units of meaning were identified and coded. The code groups underwent condensation during the refinement phase, culminating in the development of overarching thematic patterns and collective perspectives in the final synthesis. Two researchers (SH and PM) independently coded the qualitative data, and the initial intercoder agreement was assessed using Cohen’s kappa. Coding discrepancies were discussed until a consensus was reached, while a third researcher (KG) audited the ambiguous or unresolved meaning units and the development of the final themes. The final themes were grounded in the original interview material and illustrated by participant quotations in the results section. The coding process was conducted inductively, and no predefined theoretical categories related to technology acceptance were applied during coding. Instead, meaning units were grouped into descriptive themes based on the interview data. In the final synthesis, the relationships between these themes were interpreted based on existing theoretical concepts. The concept of “ambivalent acceptance” (43) was selected *post hoc* because it provided an appropriate theoretical framework to explain the coexistence of perceived benefits and practical constraints observed across the interviews.

### Data integration

Quantitative and qualitative data were integrated in accordance with a sequential explanatory mixed methods design (25). This approach aligns with the Good Reporting of a Mixed Methods Study (GRAMMS) guidelines (44, 45), emphasizing the explicit integration of diverse data strands. According to Bryman (46), “genuine integration” generates an outcome representing “more than the sum of its parts” by cross-analyzing the data strands rather than reporting them in parallel. Integration occurred at the interpretation level by establishing relationships between the quantitative and qualitative outcomes. Quantitative findings on changes in LBP, functional impairment, and quality of life were directly complemented by qualitative data describing caregivers’ experiences, enabling a contextualized interpretation of feasibility, usability, and acceptance.

### Ethics

This study was conducted in accordance with the ethical principles for research involving human participants, including the Declaration of Helsinki and applicable national regulations (47). Ethical approval for the study was obtained from the Ethics Committee of the Faculty of Medicine at Martin Luther University Halle-Wittenberg (reference number: 2024-114) prior to the initiation of data collection. The study was prospectively registered in the German Clinical Trials Register (DRKS) under registration number DRKS00036285. All participants were informed of the study objectives, procedures, potential risks, and their rights as participants. Written informed consent was obtained from all caregivers prior to their inclusion in the study. Participation was voluntary, and participants were informed that they could withdraw from the study at any time without any negative consequences. No data were collected from or analyzed for the residents of the participating facilities. Consequently, formal consent was not required from the residents. However, because exoskeletons are used in individual care situations, residents were informed about the project through written project information and, where appropriate, through direct communication. Relatives were also informed if additional information or consultation was requested. If residents or their relatives had raised objections before or during the intervention, the use of the exoskeleton in the respective care situation would have been discontinued without any disadvantage to the resident (18). No such objections were reported, and all residents accepted the implementation of the exoskeleton in their care context. Data were handled in accordance with the data protection regulations.

## Results

### Sample characteristics

All nursing staff members were invited to participate in the study at the three participating long-term care facilities. The main characteristics of the study sample are presented in Table 1. The facility-specific distribution of participants is shown in Table 2.

**Table 1:** Main characteristics.

Characteristic	Value
Initial recruited participants (*N*)	30
Included quantitative sample (*n*)	15
Included qualitative sample (*n*)	9
Female, *n* (%)	12 (80.0)
Male, *n* (%)	3 (20.0)
Age, years	28–55
Height, cm	160–180
Weight, kg	55–115

**Table 2:** Facility-specific distribution.

Facility	Initial Recruitment	Quantitative Sample	Qualitative Interviews
Bitterfeld-Wolfen	10	3	4
Witten	10	5	3
Bochum	10	7	2

A detailed assessment of participant retention revealed substantial attrition, reflecting the pragmatic challenges of longitudinal data collection in long-term care settings. Of the 30 caregivers initially recruited, 15 discontinued the 90-day quantitative data-collection phase. The documented reasons for discontinuation or incomplete participation in the quantitative data-collection phase were as follows: lack of time or forgetting to complete the required questionnaires (*n* = 8), prolonged illness lasting more than two weeks during the test period (*n* = 3), change in workplace or facility (*n* = 2), loss of study documents (*n* = 1), and withdrawal of consent (*n* = 1). This resulted in a final sample of 15 participants. No adverse events or near misses were spontaneously reported to the research team during the study period. However, because such events were not prospectively and systematically monitored, the absence of spontaneous reports cannot be interpreted as evidence that no adverse events or near misses occurred.

However, even within this cohort of 15 participants, there were missing data points. Full and complete datasets across all time points were available for only 10 individuals. Partially missing data among the remaining five participants were distributed as follows: missing second and/or third ODI questionnaires (*n* = 5), missing third SF-8 questionnaires (*n* = 2), and missing second VAS questionnaires during the intervention phase (*n* = 3).

For the subsequent qualitative phase, a purposive subsample of 9 caregivers was strategically selected from the 15 completers. Importantly, this reduction does not represent further trial attrition. Instead, it followed a standard sequential explanatory mixed-methods sampling strategy designed to capture detailed narratives from a targeted subset and provide contextual information for interpreting the observed quantitative variation.

### Quantitative results

#### Primary outcomes (VAS)

A total of 201 VAS measurements collected during the baseline phase and 111 VAS measurements collected during the intervention phase were available for 12 participants. As the measurements could not be linked at the individual level, they were analyzed descriptively as aggregated, unpaired data. The mean VAS score was 3.18 (SD = 1.34) during the baseline phase and 3.32 (SD = 1.71) during the intervention phase. Therefore, no group-level reduction in subjective pain was observed. These data were descriptively analyzed at the population level and showed no meaningful difference between the two time points (Table 3).

**Table 3:** Descriptive overview of aggregated pain-related, functional impairment, and health-related quality-of-life outcomes.

Outcome Measure	Phase	*n*	Mean	SD	Mean Difference (95% CI)	Hedges’ *g*
VAS	Pre	12	3.18	1.34	N/A (Unpaired Data)	N/A
VAS	Post	12	3.32	1.71		
ODI	T1	10	9.70	4.85	*Δ* (T3 – T1): −1.50 (−5.12, 2.12)	0.33
ODI	T2	10	9.60	5.76		
ODI	T3	10	8.20	3.58		
SF-8	T1	13	28.85	5.91	*Δ* (T3 – T1): 2.15 (−1.10, 5.40)	−0.38
SF-8	T2	13	27.85	6.56		
SF-8	T3	13	31.00	5.11		

Individual pain trajectories and within-subject changes could not be assessed because the baseline and intervention phase measurements were not paired. Therefore, the VAS findings must be interpreted strictly as descriptive group-level data and do not permit conclusions regarding individual changes in pain associated with exoskeleton use.

#### Secondary outcomes (ODI, SF-8)

The population-level outcomes for functional impairment (ODI) and health-related quality of life (SF-8) are presented in Table 3. The aggregated population data for the ODI indicated a marginal numerical decrease in functional impairment from baseline (T1: 9.70) to follow-up (T3: 8.20). However, because the cohort entered the study with a baseline mean corresponding to the “minimal disability” category (0%–20%), the capacity for measurable clinical improvement was structurally constrained by floor effects. When interpreting the SF-8 data, it is critical to note the specific scoring direction utilized in this analysis. Higher numerical scores indicate a greater degree of perceived impairment and, consequently, lower health-related quality of life. The SF-8 scores demonstrated a nonlinear trajectory, with a slight numerical decrease during the mid-study phase (T2), followed by a distinct increase at T3. Despite these overarching population trends, a descriptive examination of the individual trajectories revealed substantial heterogeneity within the cohort. This heterogeneity was evident in both the baseline values and the differing magnitudes of change over time (Figure 1). For the ODI, the cohort started with highly variable initial scores, although mean value suggested minimal disability. Notably, participants #3, #6, and #11 had lower ODI scores at the end of the study than at baseline. Conversely, the other caregivers exhibited stagnant or slightly fluctuating scores throughout the 90-day period.

**Figure 1:**
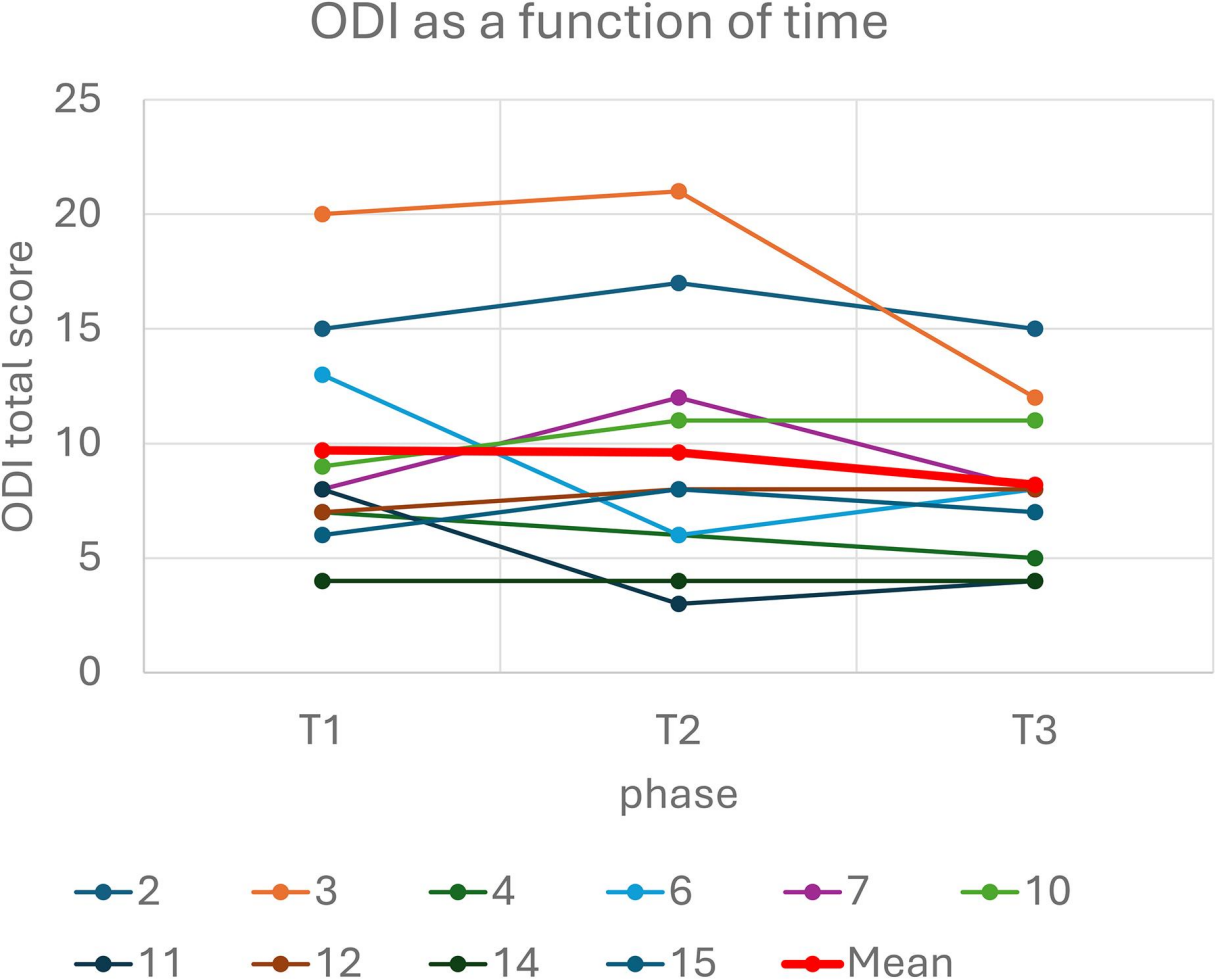
Longitudinal trajectories of functional impairment, as measured by the Oswestry Disability Index (ODI), for individual caregivers and the cohort mean across the three study phases: baseline (T1), mid-study (T2), and follow-up (T3). Lower scores indicate less functional disability. The bold line represents the mean score of the sample (*n* = 10).

Similar heterogeneity was observed in the SF-8 data. Although the overarching trend showed a V-shaped curve, the individual trajectories varied substantially (Figure 2). Some individuals showed markedly lower SF-8 scores at T2 than at baseline, corresponding to lower self-reported impairment at the measurement point before returning close to the original value at T3 (e.g., #10 and #15). However, others remained relatively stable (e.g., #1 and #9) or worsened immediately or at the end of the study (e.g., #6 and #7).

**Figure 2:**
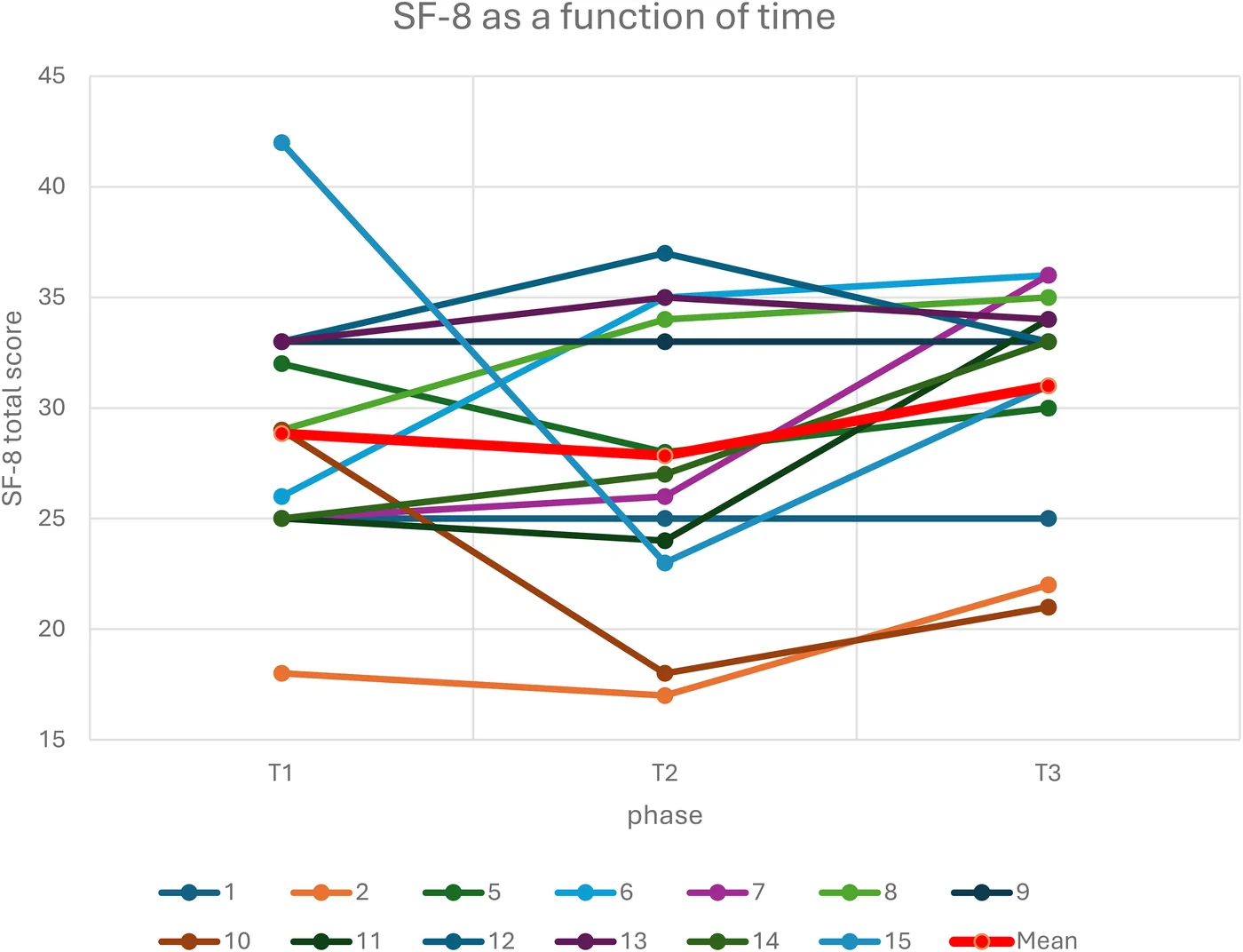
Longitudinal trajectories of health-related quality of life, as measured by the Short Form Health Survey (SF-8) overall scores, for individual caregivers and the population mean across the three study phases: baseline (T1), mid-study (T2), and follow-up (T3). Higher scores indicate a lower quality of life. The bold line represents the mean score of the sample (*n* = 13).

### Qualitative results

Nine exploratory, qualitative, topic-centered, and focused interviews were conducted (SH, KG, and SD) to capture caregivers’ subjective assessments of exoskeleton use (48–50). The participants and interviewers were neither acquainted with each other nor professionally related before the study. No company representatives were present during the interviews to ensure a confidential environment in which participants could freely express their experiences and criticisms, thereby reducing the risk of social desirability bias. The interviews were conducted for 25–30 min. The qualitative analysis, which was conducted using the STC according to Malterud (42), resulted in four overarching dimensions that described caregivers’ experiences with the use of an active exoskeleton in long-term care. These dimensions were perceived benefits, usability, barriers, and workflow integration. Across these dimensions, the findings reflect a complex pattern characterized by general openness to technological innovation, alongside substantial practical limitations. The initial inter-coder agreement was low (Cohen’s *κ* = 0.27), indicating limited concordance between the two coders during the initial independent coding. All coding discrepancies were subsequently discussed until consensus was reached, and a third researcher audited ambiguous meaning units and the final thematic structure. Subsequent themes were identified from the interview material and are illustrated in the quotations of the participants below.

#### Perceived benefits: task-specific ergonomic and postural support

A salient finding was the incongruity between the participants’ initial anticipation and their practical experience with the exoskeleton. Several caregivers initially anticipated that the device would significantly reduce their physical workload. One participant reflected:

“We really assumed, ‘Oh great, this is going to bring a real benefit.’ Our expectations were very high” (BiWoInt_4, 00:16:37).

However, these expectations differed from participants’ experiences during regular use. Rather than functioning as a substitute for physical effort, the exoskeleton is perceived as providing support only in specific care situations. The participants described the benefits of the device primarily during static forward-leaning tasks or movements involving rising from a crouched position. One caregiver explained the following:

“You quickly realize that the device was designed for lifting correctly from a squatting position. That’s where you really notice the relief… And when you're putting on compression stockings… getting back up—that’s when it helps” (BiWoInt_4, 00:21:53).

Accordingly, the perceived benefits were predominantly confined to specific care activities in which the system promoted an upright posture through trunk stabilization and supported leg-initiated movements. Conversely, during more dynamic nursing tasks requiring frequent changes in position, rapid reactions, or unrestricted mobility, the participants generally perceived the exoskeleton as offering limited benefits or interfering with their work.

#### Barriers: ergonomic ambivalence and physical discomfort

While participants reported perceived task-specific ergonomic or postural support in selected care situations, they simultaneously described several barriers that limited their overall perceived benefits. A recurrent theme that emerged from the interviews was that the intended relief was accompanied by the emergence of a new physical burden. One caregiver provided the following testimony:

“There is already a certain amount of additional weight. And if you wear it for several hours, you really notice that extra pressure on your body” (BoInt_2, 00:10:15).

Rather than entirely eliminating physical strain, the exoskeleton was perceived as shifting part of the load to other body regions. Participants further described the structural design of the device as insufficiently compatible with the confined spatial conditions characteristic of long-term care. One participant explained the following:

“You're simply much wider. When I'm working in a resident’s room and space is limited, I keep bumping into everything. That actually destroys the ergonomic movement because I have to twist my body just to avoid getting caught” (BiWoInt_ 2, 00:15:20).

In such situations, the caregivers reported compensatory movements that counteracted the intended postural support provided by the device. Similar experiences were described for highly dynamic care activities, during which the device occasionally interfered with natural movement patterns and increased, rather than reduced, physical effort. Moreover, several participants identified heat accumulation and perspiration beneath the device as additional sources of discomfort, which, in some cases, resulted in the premature removal of the exoskeleton despite its perceived supportive function.

#### Operational usability: learning requirements and routine integration

A close relationship emerged between usability and the caregivers’ ability to handle the exoskeleton with confidence in their routine practices. Participants repeatedly asserted that safe and efficient use necessitated not only an initial introduction but also repeated practice to cultivate familiarity with the system. As one participant explained: “you really need time to get used to it. You can't say after three days that you've mastered it” (BiWoInt_3.1, 00:02:08). This account reflects the participants’ perception that familiarity with the device developed gradually rather than immediately. However, the learning curve was not quantified, and the study did not collect objective longitudinal indicators such as donning or adjustment time, task-specific proficiency, or frequency of use over time. Furthermore, the participants reported a decline in their knowledge of operating the device when it was not regularly used. This finding underscores the need for readily available support for daily work activities. One caregiver articulated challenges as follows:

“..you simply forget it. Which button was the right one? How do I charge it now? I'd really like to have a written, illustrated instruction guide” (WiInt_1, 00:14:09).

Furthermore, the process of fitting and adjusting the exoskeleton was repeatedly described as time-consuming, particularly in situations involving time-critical nursing routines or when the device was shared among multiple users. The findings indicate that the successful implementation of wearable assistive technologies is contingent not only on intuitive technical design but also on sustained opportunities for learning, simplified adjustment procedures and immediate access to operational guidance.

#### Workflow integration: selective and context-dependent use

The integration of exoskeletons into quotidian nursing workflows was consistently described as being highly context-dependent. Participants reported using the exoskeleton selectively during physically demanding activities involving prolonged static postures or lifting maneuvers rather than continuously throughout an entire shift. As one caregiver explained, “the device is meant to provide support […] you use it as an assistive tool” (BiWoInt 4, 00:03:48), while simultaneously emphasizing that it should only be worn temporarily because prolonged use became uncomfortable and therefore unsuitable for an entire shift. The participants indicated that the suitability of the exoskeleton was contingent on task characteristics. One participant succinctly encapsulated this selective applicability by noting that the device functioned effectively when lifting heavy objects, such as a crate of water, while “when working with the patient […] it was rather not so good” (BoInt 1, 00:02:46). The integration of the device into routine nursing practice was further constrained by the following three factors: First, time constraints are inherent to the nursing process. Second, effort is required for donning and adjusting devices. Third, nursing workflows are dynamic. Consequently, the participants described the exoskeleton not as a replacement for established work practices but as a situational assistive technology whose use depended on the specific care activity and organizational context.

#### Synthesis: balancing perceived benefits and practical constraints

The participants consistently described both enabling and constraining experiences with the active exoskeleton across all four qualitative dimensions. Caregivers expressed openness toward technological innovation and recognized the system’s potential to support occupational health, especially during physically demanding and static care activities. However, they also reported practical limitations related to ergonomics, usability, and workflow integration, which they perceived as barriers to routine use. Although they generally perceived the system’s basic functionality to be intuitive, the participants emphasized the need for future developments that focus on reducing device weight, improving anthropometric fit, increasing adaptability to different body types, and enabling faster and more individualized adjustment procedures. Participants described the strap system, fitting process, and time required for individual adjustments as barriers to the seamless integration of the device into everyday nursing practice. The findings indicated a recurring pattern in which caregivers balanced perceived task-specific ergonomic or postural support against the practical limitations encountered during their daily nursing work. This pattern is evident in the findings and contrasts with more straightforward expressions of expected acceptance or rejection.

Lucke’s (43) concept of ambivalent acceptance is used to interpret this empirical pattern. This concept provides an established theoretical framework for understanding how positive evaluations of a technology can coexist with reservations arising from its practical implementation in everyday life. Participants’ accounts suggest that their acceptance of the active exoskeleton was associated not only with its perceived functional benefits but also with its perceived compatibility with the organizational and workflow conditions of long-term care.

### Integrative overview

The primary objective of this exploratory pilot study was to examine the implementation of an active exoskeleton across four complementary dimensions: feasibility, usability, caregiver acceptance, and descriptive pain-related outcomes. Quantitative measures also provided preliminary descriptive parameters for planning future confirmatory studies. To ensure comprehensive and GRAMMS-compliant integration of the different data strands, Table 4 systematically links all collected quantitative dimensions with the central qualitative themes identified in this study (45). By considering each quantitative outcome (VAS, ODI, and SF-8) alongside the corresponding qualitative themes, this integrative approach facilitates mutual illumination and provides contextual information for interpreting convergence and divergence between the data strands (46, 51).

**Table 4:** Integration of the mixed-methods results (quantitative results and qualitative narratives).

Quantitative dimension	Observed results and trajectories	Corresponding qualitative dimension (narratives)	Integrative focus (mutual illumination)
Subjective pain (VAS)	**No relevant descriptive population-level change:** Mean scores remained virtually stable between the baseline and intervention phases. The aggregated, unpaired VAS data showed no group-level reduction in subjective pain. Individual pain trajectories could not be assessed.	**Situational support vs. expectation-reality gap:** Participants reported localized relief but also novel physical burden in daily practice.	Participants reported perceived localized relief or task-specific postural support during selected high-load activities; however, this was not reflected in a group-level reduction in aggregated unpaired VAS data. Individual pain trajectories could not be assessed.
Functional impairment (ODI)	**Individual changes in ODI scores:** Participants #3, #6, and #11 had lower scores at follow-up than at baseline. The cohort mean changed from 9.70 at T1 to 8.20 at T3.	**Benefits in static care:** Narratives emphasize the stabilizing effect and perceived postural support during prolonged forward-leaning activities.	Lower ODI scores in some participants were considered alongside qualitative reports of perceived task-specific postural support during static care activities. In light of the exploratory design, it is not possible to infer a causal relationship between these findings.
	**Structural floor effect:** Most participants started with “minimal disability” in the ODI (9.70 baseline mean).	**Selective workflow integration:** Participants described time-consuming donning and doffing and spatial constraints as barriers to routine use and reported a preference for using the device during selected high-exertion tasks.	The device was introduced as an ergonomic assistive technology rather than as a rehabilitative intervention. Participants reported that they considered it particularly applicable to selected physically demanding tasks.
Quality of life (SF-8)	**V-shaped curve/deterioration:** After a slight mid-study improvement, scores often increased by T3, sometimes even above baseline levels (e.g., #2, #5).	**Ergonomic ambivalence & barriers:** Reports of secondary burdens, including device weight, shoulder strain, thermal discomfort (heat/sweat), and restricted mobility in confined spaces.	Heterogeneous SF-8 trajectories were observed in conjunction with qualitative reports of device weight, shoulder strain, thermal discomfort, and restricted mobility. These findings describe concurrent experiences but do not establish that device-related burdens caused the observed SF-8 trajectories.

Overall, the integrated findings suggest a recurring tension between perceived task-specific postural support and practical, ergonomic, and workflow-related factors that participants described as barriers to sustained use. Caregivers’ responses did not indicate straightforward acceptance or rejection. Rather, caregivers appeared to weigh the perceived ergonomic benefits against additional physical, temporal, and organizational burdens in a situational and pragmatic manner. The integrated pattern resulting from this process provides a foundation for a theoretical interpretation of acceptance, as presented in the following discussion.

## Discussion

The present findings provide a new perspective on the feasibility, usability, descriptive pain-related outcomes, and acceptance of active exoskeleton use in long-term care. Across all four evaluation dimensions–feasibility, usability, descriptive pain-related outcomes, and acceptance–the findings consistently point toward a pattern of ambivalent acceptance (43), in which participants’ reports of perceived task-specific postural support coexisted with their accounts of ergonomic, organizational, and workflow-related barriers to sustained integration into everyday nursing practice. It is crucial to note that these findings reflect the *subjective* experiences and perceived usefulness reported by caregivers, as the study design did not incorporate objective kinematic or physiological measures (such as EMG or metabolic cost analysis) to definitively confirm mechanical effectiveness.

Regarding feasibility, the participants described exoskeleton use as highly context-dependent and shaped by organizational, environmental, and task-related factors, supporting previous implementation-oriented research and highlighting the importance of the work setting, task suitability, and workflow compatibility for sustained exoskeleton use in healthcare environments (52). Specifically, the time required to don and doff a device was frequently perceived as disproportionate to the duration of many care activities. As demonstrated in previous studies, in high-pressure care scenarios, supplementary temporal demands have been shown to decrease the probability of digital device utilization, particularly for brief or uncertain tasks (53, 54).

Participants described the dimensions of the device as problematic in confined environments and reported adapting their movements or, in some situations, refraining from using the device. These constraints are particularly salient in dynamic care situations requiring frequent repositioning, rapid responses, or close physical interactions with residents. This qualitative feedback provides contextual information for interpreting the divergent quantitative trajectories: while participants reported perceived task-specific ergonomic or postural support of the exoskeleton, the interview accounts suggest that caregivers perceived a tension between task-specific support and the practical challenges and secondary physical burdens associated with device use. The participants’ reports indicated no consistent improvement in their work-related quality of life. However, they also reported experiencing additional strains during routine tasks. Furthermore, the HAL® Lumbar Type was originally developed within a Japanese context and may therefore not fully reflect the body proportions and movement patterns of German nursing staff (14, 20). Another aspect of the qualitative interviews was the fit and adjustability of the system. Although the technical fit was examined for each participant before utilization, participant-level anthropometric characteristics, device size allocation, and specific fitting problems were not systematically documented. Consequently, the anthropometric fit could not be analytically evaluated, and the present data do not allow for conclusions as to whether the reported adjustment difficulties were associated with the body dimensions of the participants or the available device sizes. Subsequent studies should systematically document the anthropometric measurements, device size allocation, and fitting issues. This will allow the examination of their potential relationships with usability, comfort, and acceptance. Limited adjustability may amplify discomfort, restrict mobility, and reduce acceptance in everyday care practices. These findings highlight the importance of integrating broader anthropometric variability and context-specific workflow requirements into future exoskeleton developments, particularly when technologies are intended for international implementation. Participants’ accounts suggest that perceived secondary strain and usability problems may discourage routine use. The qualitative findings suggest a perceived “context gap” between the device’s intended technical use and the dynamic reality of long-term care, particularly in relation to reported spatial constraints and temporal demands. Participants frequently described the structural bulk of the HAL® system as incompatible with the confined environments of residents’ rooms, impeding natural and adaptive movement patterns, particularly in dynamic situations requiring rapid reaction or close physical interaction. To compensate for these spatial limitations, caregivers reported compensatory movements that they perceived as counteracting the device’s intended postural support and increasing their physical effort (4). Participants perceived the time required for donning, adjustment, and doffing as a barrier to feasible use within routine care workflows. Specifically, the time required for donning and doffing was perceived to be disproportionate to the duration of many episodic nursing activities. Similar observations have been reported for robotic mobilization systems, where prolonged “setup times” appear to offset perceived mechanical advantages in routine care practice (54). These findings indicate that biomechanical evaluations under controlled or task-specific conditions are inadequate. Participants’ accounts suggest that perceived usability and willingness to use the device may be associated with its compatibility with workflows, environmental conditions, and time constraints (52). This interpretation aligns with recent systematic reviews emphasizing that exoskeleton research predominantly relies on laboratory-based assessments, whereas real-world workplace evaluations remain comparatively scarce (55). These findings emphasize the necessity for subsequent studies to assess both the clinical effectiveness and circumstances under which exoskeletons can be feasibly integrated into standard care. The qualitative accounts provide contextual information that may assist in interpreting the heterogeneous quantitative trajectories.

The heterogeneous individual trajectories in functional impairment and health-related quality of life were considered alongside qualitative reports of ergonomic ambivalence and perceived barriers to routine integration. The qualitative accounts provide contextual information but cannot explain the causes of the quantitative trajectories. The higher mean SF-8 score at T3, indicating lower self-reported health-related quality of life at that measurement point, occurred alongside qualitative reports categorized as “ergonomic ambivalence” and “physical discomfort.” No causal relationship between these findings can be inferred. Caregivers who frequently performed highly dynamic tasks in confined spaces reported frustration, movement restriction, and secondary shoulder pain owing to the weight of the device. Our findings reveal a key “ergonomic ambivalence” (12) that is consistent with other research findings on passive exoskeletons, where perceived task-specific support and lower ODI scores in some participants coexisted with reports of secondary physical burdens (16, 56). These challenges primarily included the device weight of 3.1 kilograms. Participants associated this weight with perceived secondary strain and reported thermal discomfort and heat accumulation during use, as described in a similar way when using passive exoskeletons (13). Participants describe these factors as barriers to continuous device use. Lower ODI scores in some participants did not coincide with consistently more favorable SF-8 trajectories. Qualitative accounts provide contextual information about discomfort and implementation barriers but cannot establish the causes of these divergent patterns. In this study, nurses reported a preference for using the exoskeleton during static tasks, such as bedside care, while perceiving it as a hindrance in dynamic or time-critical care scenarios characterized by frequent movement changes and confined spatial conditions. This observation can be interpreted as a rational domestication strategy (12, 53, 54). They assess the mechanical benefit in relation to the physical discomfort (e.g., heat accumulation and shoulder strain) and the “script” of the device, which often conflicts with the dynamic reality of nursing, namely “usability” (57).

Rather than necessarily indicating implementation failure, participants’ accounts may be interpreted as consistent with a process of pragmatic domestication (57). Domestication is defined as the process by which users actively “tame” and integrate technology into their specific everyday routines based on a situational assessment of its utility (58, 59). In this strategy, expert users navigate the trade-off between physical support and operational serviceability in a situational manner. Accordingly, the pattern of “ambivalent acceptance” should be interpreted as reflecting the active role of caregivers as expert designers of their workflows. These caregivers domesticate the HAL® system to fit specific socio-technical arrangements while maintaining their professional agency in others. In this sense, participants’ reported reluctance to use the device routinely may reflect, at least in part, a pragmatic response to an unfavorable balance between perceived relief and newly introduced burdens. This insight contributes to addressing the “dilemma behind the product” and underscores the importance of designing assistive systems that minimize secondary strain while preserving or enhancing their primary supportive function.

These patterns of ambivalent acceptance can be interpreted through Lucke’s conceptualization of acceptance as a subjective, social, and situationally negotiated form of legitimacy (43). This refers to an interest-driven acceptance, whereby legitimacy is not taken for granted but remains a “subjective-social” construct that depends strictly on a reflexive assessment of individual interests and the successful “reappropriation” of the technology within the user’s specific everyday context. The concept of acceptance in this context is not driven solely by perceived effectiveness; rather, it appears to be shaped by a balance between the benefits derived from a given practice and the effort required to implement it within the constraints of everyday practices. This underscores the necessity for future technological developments to prioritize not only biomechanical performance but also seamless integration into real-world care processes. Caregivers’ accounts suggest that perceived feasibility was associated with the relationship between the device’s technical usability and the specific demands of nursing workflows (22). From a Technology Acceptance Model (TAM) perspective (60–63), participants’ accounts may be interpreted as reflecting a tension between perceived ease of use and perceived usefulness. Therefore, the participants’ accounts may be interpreted as consistent with a process of pragmatic domestication. Rather than indicating a failure of implementation, participants’ reports of selective use, including a perceived preference for static and predictable tasks over dynamic or unpredictable activities, may reflect a pragmatic assessment of the device’s task-specific suitability. Expert users navigated the trade-off between perceived task-specific support and operational efficiency to maintain their professional agency within the complex realities of long-term care.

The aggregated unpaired VAS data showed no group-level reduction in subjective pain. As measurements could not be linked at the individual level, individual pain trajectories and within-subject changes could not be assessed. This finding is notable because participants reported perceived task-specific postural support during specific high-load activities. This finding aligns with previous research that demonstrated that exoskeletons may offer perceived task-specific physical or postural support without necessarily resulting in measurable reductions in the overall pain trajectories or muscle activation patterns (56). In this context, selective and task-specific use of the device appears to be particularly relevant. Consistent with the observations reported by Exner et al. (12), nurses reported predominantly using the exoskeleton during selected high-load tasks, such as lifting or repositioning residents, rather than continuously throughout their shifts. No relationship between device exposure and pain could be examined because the wearing time and task-specific use were not systematically recorded. A notable “expectation-reality gap” was identified, indicating that initial anticipations of immediate and effortless pain relief, often shaped by “science fiction”-like imaginaries surrounding exoskeleton technologies, diverged from the practical reality of everyday use (64). Rather than functioning as an autonomous substitute for physical effort, the device requires active adaptation and provides targeted, rather than universal, support. As indicated by Exner et al. (12), analogous incongruities between anticipated and experienced effects have been documented in previous field studies. In these studies, participants reported that their perceived level of assistance was lower than initially anticipated. Brandt (16) similarly underscored that excessively optimistic expectations may contribute to a negative perception of effectiveness when practical support does not fully align with the initial assumptions.

A relevant consideration when interpreting the ODI trajectories is the potential floor effect resulting from the low mean baseline score. The cohort entered the study with a mean ODI score of 9.70, which falls into the category of “minimal disability” (0%–20%). Given that the established MCID recommendations for the ODI (65) indicate improvements of 6–15 points for clinical relevance, achieving such a reduction was mathematically and practically unattainable for this relatively healthy group. The exoskeleton was introduced in this study as an ergonomic assistive technology for routine nursing work rather than as a rehabilitative intervention. This interpretation aligns with the prevailing evidence suggesting that the occupational risk of LBP in nursing is predominantly influenced by cumulative peak loads associated with manual handling and sustained non-neutral postures, rather than by routine ambulatory activity. The qualitative findings indicate that participants perceived task-specific postural support during physically demanding care activities. However, the quantitative data do not provide evidence of biomechanical or postural effects.

### Strength and limitations

This study had several merits. First, it evaluated the use of actively controlled exoskeletons in real-world long-term care settings, thereby addressing a notable gap in existing research that has predominantly focused on passive systems or laboratory-based conditions. The evaluation of the device within real-world long-term care settings over a 90-day intervention period enhanced the ecological relevance of the findings and provided insights into its practical applicability beyond short-term or simulated use. Furthermore, the investigated system exemplifies an active sensor-based assistive technology that responds to users’ bioelectrical signals and provides adaptive support. In contrast to numerous passive exoskeletons, which depend principally on mechanical structures such as springs, bands, or harness systems, active systems involve dynamic human–machine interactions. This facilitates not only the assessment of biomechanical effects but also the evaluation of usability, acceptance, and interaction processes in real-world care settings. From this perspective, this study contributes to a more comprehensive understanding of technology-supported care and extends existing evidence beyond purely mechanical support systems.

A potential limitation concerns the involvement of the manufacturer’s staff during the initial device training. To mitigate any influence on the participants’ responses, we maintained a strict separation between technical support and scientific evaluation. Cyberdyne had no access to the raw data, qualitative transcripts, or results at any time during the study period. Furthermore, the use of interviewers who were not part of the initial training process helped ensure an independent evaluative relationship with participants. Second, the utilization of a mixed-methods design constitutes a significant strength. The integration of quantitative outcome measures with qualitative interview data provided a more comprehensive description of the outcome trajectories and contextual factors associated with implementation. Concurrently, instances of divergence were observed, including instances in which more favorable quantitative scores at some measurement points coincided with negative qualitative evaluations. These divergences were not interpreted as evidence of a causal relationship between the data strands. Instead, they were used to illustrate that the quantitative measures and qualitative interviews captured different aspects of participants’ experiences. Alternative explanations, including measurement limitations and the absence of objective exposure data, cannot be excluded.

However, when interpreting these findings, it is imperative to consider their limitations. This study employed a non-randomized exploratory design devoid of a control group, a limitation that hinders the ability to derive causal inferences concerning the effects of the intervention. Accordingly, the present pilot study does not allow for inference of the effectiveness of the intervention or its potential preventive effects on work-related musculoskeletal disorders. The implementation of a snowball sampling strategy might have engendered selection bias, potentially favoring institutions and individuals with a higher proclivity for innovation or prior research involvement. Additionally, the relatively modest sample size and voluntary nature of participation may have introduced selection bias, thereby limiting the generalizability of the findings. The primary objective of this pilot study was to assess the feasibility, usability, and acceptance of an exoskeleton under routine care conditions. In accordance with the published study protocol, participants were instructed to use the exoskeleton during physically demanding work periods and care activities. However, objective exposure and utilization data were not systematically collected. Individual wearing time, the number of shifts during which the device was used, task-specific use, overall frequency of use, work-cycle structure, and the frequency of donning and doffing were not recorded. Consequently, adherence to the intended implementation and actual utilization patterns could not be objectively assessed. Statements regarding selective use, task suitability, feasibility, and workflow integration are therefore based primarily on participants’ qualitative accounts and should be interpreted as participant-reported implementation experiences rather than objectively verified utilization patterns. The quantitative usability measures do not provide objective information about the frequency, duration, or context of device use. This study did not incorporate objective biomechanical or ergonomic workload metrics. Consequently, the ergonomic and postural support reported in this study is indicative of the participants’ task-specific perceptions and should not be interpreted as evidence of objectively measured biomechanical benefits. Another limitation pertains to the anthropometric fit of the device. Despite the efforts of trained facilitators to ensure proper positioning and technical compatibility of the exoskeleton before use, there is a notable absence of systematic documentation concerning participant-level BMI, waist circumference, device size allocation, and the occurrence of specific fitting problems. Consequently, the anthropometric fit of the participants was not the focus of the analytical evaluation, which precluded the determination of any potential associations among the body dimensions of the participants, the size of the device, the degree of difficulty experienced during fitting, and the level of perceived usability. The quantitative pain assessment was constrained by the inability to consistently link pre- and post-intervention VAS measurements at the individual level, which is a limitation stemming from the demands of routine care shifts. This precluded within-subject analyses and restricted the evaluation of pain-related outcomes to descriptive analyses of aggregated group-level data. However, the structural failure of fine-grained, shift-by-shift VAS documentation represents a critical feasibility finding in itself. Despite the initial engagement, the ongoing collection of repeated subjective measurements could not be sustained under real-world conditions owing to staff shortages, sickness absence, vacation periods, shift-related workloads, and unforeseen operational demands. Adverse events and near misses were not prospectively and systematically monitored using a standardized instrument or dedicated event log. The available information was limited to events spontaneously reported by participants through their contacts with the research or technical support teams. Therefore, the absence of spontaneous reports does not permit conclusions regarding the occurrence or frequency of adverse events and near misses or the safety of the device under routine care conditions. Future efficacy trials in long-term care must completely decouple data collection from standard nursing duties by relying on permanently assigned on-site research coordinators to ensure data integrity without exacerbating occupational burdens. In contrast to the ODI and SF-8, which require assessment only at predefined time points, repeated pre- and post-shift VAS measurements generate a substantially higher documentation burden in routine practice. These findings suggest that future longitudinal implementation studies in nursing settings should integrate dedicated onsite research support such as study nurses or permanently assigned research coordinators at the project planning stage. The necessity of such support structures is twofold: first, to ensure the reliability of data collection and adherence to established protocols; and second, to alleviate the additional organizational demands placed on nursing staff and ward management in care facilities that are increasingly engaged in participatory technology evaluation projects. Qualitative findings, while providing valuable contextual insights, are based on a purposive subset of participants and may not fully represent all perspectives within the study population. Moreover, the prioritization of qualitative explanations in the context of heterogeneous quantitative results entails the potential for a disproportionate influence on the overall interpretation. The initial inter-coder agreement was low (Cohen’s *κ* = 0.27), indicating limited concordance between the two coders during the initial independent coding. This represents a methodological limitation and may have affected the dependability of the initial coding process. All coding discrepancies were subsequently examined through a structured consensus process, and a third researcher audited ambiguous meaning units and the final thematic structure. These procedures enhanced the transparency and reflexivity of the analysis but do not eliminate the limitation associated with the low initial agreement. The qualitative findings should therefore be interpreted with appropriate caution. Finally, the qualitative data were collected in German and translated into English for reporting. Despite meticulous efforts to maintain the integrity of the original meaning, the translation process may have introduced subtle variations in interpretation (translation bias). These limitations underscore the exploratory nature of this study, while the combination of real-world implementation and mixed-methods analysis provides insights into the feasibility, usability, and acceptance of active exoskeletons in long-term care (39). However, it is imperative to exercise caution and ensure balanced integration of data strands to avoid overreliance on a single source of evidence.

### Implications

These findings suggest that active lumbar exoskeletons may provide perceived task-specific ergonomic or postural support during selected tasks involving high physical demands or sustained non-neutral postures. However, their routine use necessitates meticulous integration into existing workflows, emphasizing the identification of task-specific use cases as opposed to presuming uninterrupted applicability across entire shifts. From a design perspective, the identified barriers underscore the need for lighter, more compact, and more intuitive systems. The enhancement of usability and comfort, particularly through weight reduction, minimization of thermal burden, and improvement of fit, is a critical prerequisite for broader acceptance. Moreover, future developments must prioritize compatibility with the spatial constraints and dynamic work processes inherent in care environments. In a broader sense, this study underscores the necessity of evaluating assistive technologies in nursing that extend beyond biomechanical outcomes to incorporate the contextual and experiential factors that shape their real-world use.

Thus, future research should prioritize the implementation of longitudinal real-world studies and the integration of robust designs that incorporate individual-level outcome tracking. The utilization of randomized controlled trials is imperative for evaluating the causal effects on physical strain, pain reduction, and work-related health outcomes. To complement self-reported data, future studies should incorporate objective biomechanical and physiological measurements, such as electromyography, to quantify muscle activation patterns and the precise extent of load reduction during specific care activities. In addition, task-specific ergonomic exposure should be assessed using appropriate standardized observational methods, such as the Rapid Entire Body Assessment (REBA) (66), Rapid Upper Limb Assessment (RULA) (67), and Ovako Working Posture Analysis System (OWAS) (68). These methods should be complemented by time-motion analysis and inertial measurement unit sensors to quantify trunk angles and movement patterns. Future studies should include task-exposure logs, objective records of wearing time and task-specific use, measurements of donning, adjustment, and doffing time, and systematic anthropometric fit assessments documenting body dimensions, device size allocation, and fitting-related difficulties. Objective measures of this type would facilitate a more nuanced understanding of the mechanisms of technology and support a more differentiated evaluation of its effectiveness. Moreover, larger sample sizes and individual-level longitudinal data are necessary to capture the variability in user responses more accurately and to assess within-subject changes over time. Research should also be extended to diverse care settings encompassing acute care and ambulatory services, given the substantial variation in physical demands and workflow structures across these settings. Finally, comparative studies encompassing both active and passive exoskeleton systems across various use cases are of significant value. The implementation of such research endeavors could facilitate the identification of the most suitable system for specific occupational demands and care contexts, ultimately supporting the development of targeted and effective implementation strategies in nursing practice.

## Conclusion

This exploratory, mixed-methods, real-world feasibility and implementation study identified a range of responses to the HAL® Lumbar Type in long-term care. The participants generally found that the exoskeleton offered ergonomic or postural support during specific activities that required considerable physical effort. At the same time, they reported that they generally did not consider the device suitable for continuous use throughout a full work shift under the conditions they experienced. Their accounts suggest that the perceived feasibility and willingness to use the device depended not only on its technical functionality but also on its compatibility with the realities of care work. The relevant conditions that were considered included task specificity, workflow compatibility, the time and effort required for donning, adjustment, and doffing, device weight, thermal discomfort, spatial constraints, and perceived fit and adjustability. The aggregated unpaired VAS data did not indicate a group-level reduction in subjective pain, and individual pain trajectories could not be assessed. It is important to note that the descriptive ODI trajectories were heterogeneous, with lower follow-up scores for some participants. These ODI findings were considered alongside qualitative reports on perceived task-specific postural support during selected care activities. However, it is important to note that no causal relationship between these findings can be inferred. Therefore, this study did not provide evidence of the clinical effectiveness, biomechanical improvement, ergonomic risk reduction, or prevention of musculoskeletal disorders. Finally, although caregivers showed high initial acceptance and openness toward the technology, their reported willingness to adopt the system over the long term remained pragmatic. This willingness appeared to depend on a favorable balance between perceived task-specific support and the additional effort required for daily operations. Notably, long-term adoption was not measured after the completion of the intervention. Therefore, the interpretation was based on the participants’ qualitative accounts and practical experiences during the 90-day study period. The primary contribution of this study was to identify nurse-reported factors that may influence perceived acceptability and integration of an active lumbar exoskeleton into routine long-term care. In the future, it would be beneficial to conduct controlled longitudinal studies that examine the possible preventive effects using objective utilization and biomechanical measures. These studies should also assess sustained adoption beyond the intervention period. In the future, we should consider ways to make devices lighter, more comfortable to touch, easier to handle, and compatible with the changing and sometimes limited spaces where caregiving takes place.

## Data Availability

The datasets presented in this article are not readily available because participants did not provide consent for their data to be shared with third parties, and the ethics approval obtained for this study does not cover data sharing beyond the research team. In addition, the qualitative interview data and individual-level health data cannot be fully anonymised given the small sample size and the limited number of participating facilities. Aggregated data are reported in the article. Requests to access the datasets should be directed to Sebastian Hofstetter, sebastian.hofstetter@medizin.uni-halle.de.
